# The complete mitogenome of *Schizopygopsis stoliczkai* (Cypriniformes: Cyprinidae) from Western China

**DOI:** 10.1080/23802359.2016.1219630

**Published:** 2016-09-04

**Authors:** Chao Zhang, Liqiang Chen, Chengzhi Ding

**Affiliations:** Yunnan Key Laboratory of International Rivers and Transboundary Eco-Security, Yunnan University, Kunming, China

**Keywords:** The Tibetan Plateau, schizothoracine, phylogenetic relationships

## Abstract

In this study, we firstly sequenced the complete mitochondrial genome of *Schizopygopsis stoliczkai* (Cypriniformes: Cyprinidae) from the Yarkand River, Xinjiang, China. The mitogenome is 16,833 bp in length, composed of 13 protein-coding genes, 22 transfer RNA genes, 2 ribosomal RNA genes, and 2 non-coding regions (origin of light-strand replication and control region). The gene content and order is in accord with the common vertebrate form. The nucleotide base composition of H-strand is 28.4% A, 27.0% T, 26.1% C, and 18.5% G. The phylogenetic analysis based on the complete mitochondrial sequences showed that *S. stoliczkai* was clustered into *Schizopygopsis* clade.

The schizothoracine fish is the largest and the most diverse group of ichthyofauna on the Tibetan Plateau (Chen & Cao 2000; He & Chen 2006), which was divided into three evolutionary grades: primitive, specialized, and highly specialized fishes (Cao et al. 1981). *Schizopygopsis stolickai* Steindachner 1866 is the westernmost distributed species of the highly specialized grade schizothoracines, and widely distributed in the western Tibetan Plateau, Pamir Plateau, and Helmand River in Central Asia (He & Chen 2007). In local rivers and lakes, *S. stoliczkai* normally acts as the dominant fish species and plays a crucial role in commercial catches. However, impeded by harsh natural condition, molecular studies about *S. stoliczkai* are still very limited, only a few phylogeny studies based on several sequences of mitochondrial genome were conducted (He & Chen 2007; He et al. 2016). In this study, for the first time we sequenced the complete mitochondrial genome of *S. stoliczkai* (GenBank accession no. KX443413), which would be useful for further genetic studies, phylogenetic analysis, and conservation of this species.

The sample of *S. stoliczkai* was collected from the Yarkand River, a tributary of Tarim River in Pishan County (N36°19′25″, E78°6′14″), Xinjiang, Western China. The specimen was stored in Institute of Hydrobiology, Chinese Academy of Science (voucher number IHB160623). Whole genomic DNA was extracted using the standard phenol–chloroform method (Sambrook et al. 1989). To obtain the whole mitochondrial sequence of *S. stoliczkai*, we designed 16 pairs of primers with reference to the mitochondrial sequence of *Schizopygopsis anteroventris* (Liang et al. 2016). Sectioned sequences were aligned with Bioedit v. 7.2.5(http://www.mbio.ncsu.edu/bioedit/bioedit.html) and then the nucleotide composition was calculated using soft MEGA version 6.0 (Tamura et al. 2013).

The complete mitogenome of *S. stoliczkai* is 16,833 bp in length, including 13 protein-coding genes, 22 transfer RNA (tRNA) genes, 2 ribosomal RNA (rRNA) genes, and 2 noncoding regions: origin of light-strand replication (OL) and control region (D-loop). The gene content and order are identical with the common vertebrates (Miya et al. 2001). Except for a protein coding gene (*ND6*) and eight tRNA genes (*tRNA^Gln^*, *tRNA^Ala^*, *tRNA^Asn^*, *tRNA^Cys^*, *tRNA^Tyr^*, *tRNA^Ser^*, *tRNA^Glu^,* and *tRNA^Pro^*) that encoded on the light-strand (L-strand), most genes are encoded on the heavy strand (H-strand). The overall nucleotide composition of H-strand is 28.4% A, 27.0% T, 26.1% C, and 18.5% G, showing a slight A + T-rich feature (55.4%) as observed in most teleost fishes (Wang et al. 2013). A total of nine overlaps are detected, with four among the 13 protein-coding genes (*ATP8*-*ATP6*, *ATP6*-*COXIII*, *ND4*-*ND4L*, and *ND5*-*ND6*), two among the 22 tRNA genes (*tRNA^Ile^*-*tRNA^Gln^*, *tRNA^Cys^*-*tRNA^Tyr^*) and three overlaps in the pairs of genes *ND2*–*tRNA^Trp^*, *COXIII*–*tRNA^Gly^*, and *ND3*–*tRNA^Arg^*.

Based on complete mitochondrion sequence, the phylogenetic tree of *S. stoliczkai* with 13 closely related species was constructed using maximum-likelihood (ML) analysis with the General Time Reversible model, Gamma distributed with Invariant sites, Nearest-Neighbour-Interchange (NNI) and 1000 bootstrap replicates in soft MEGA version 6.0 (Tamura et al. 2013). *Cyprinus carpio* was used as outgroup. The mtDNA sequences of this 14 species were downloaded from GenBank. The ML tree was shown in Figure 1. The log-likelihood score for the ML tree was −72221.42. The phylogenetic tree, supported by high bootstrap, showed that *S. stoliczkai* has close affinities with other genus *Schizopygopsis* species and each genus species formed an independent cluster.

**Figure 1.**
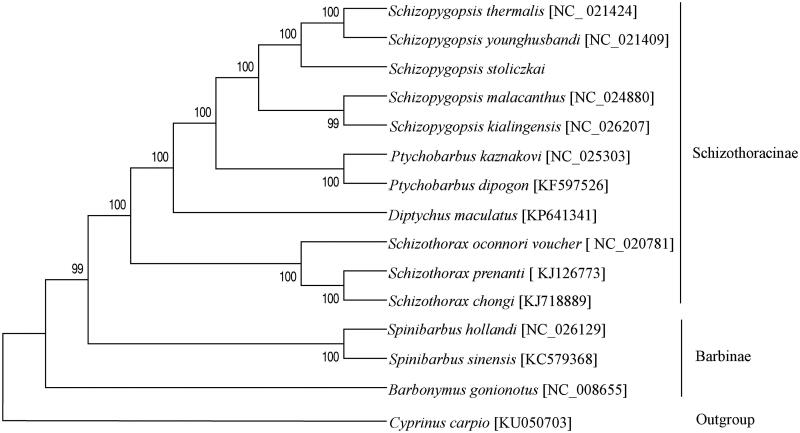
The consensus phylogenetic relationship of *S. stoliczkai* with 13 closely related species. Bootstrap support is indicated at nodes. GenBank accession numbers are indicated in brackets.
